# Diagnostic Accuracy of Sternum Measurements for Sex Estimation: A Systematic Review with Meta-Analysis

**DOI:** 10.3390/diagnostics16091255

**Published:** 2026-04-22

**Authors:** George Triantafyllou, Daniel Gondorf, Ioannis Paschopoulos, Eric Baccino, Laurent Martrille, Stavroula Papadodima, Maria Piagkou

**Affiliations:** 1 Department of Anatomy, School of Medicine, Faculty of Health Sciences, National and Kapodistrian University of Athens, 11527 Athens, Greece; georgerose406@gmail.com (G.T.); daniel.gondorf@gmail.com (D.G.); johnpascho@gmail.com (I.P.); mapian@med.uoa.gr (M.P.); 2 EDPFM, Department of Legal Medicine CHU Montpellier, University of Montpellier, 34000 Montpellier, France; e-baccino@chu-montpellier.fr (E.B.); laurent.martrille@chu-montpellier.fr (L.M.); 3 Department of Forensic Medicine and Toxicology, School of Medicine, Faculty of Health Sciences, National and Kapodistrian University of Athens, 11527 Athens, Greece

**Keywords:** sternal measurements, sex estimation, diagnostic accuracy, forensic anthropology, meta-analysis

## Abstract

**Background**: Sex estimation represents a pivotal element of forensic anthropological investigation, conventionally dependent on highly dimorphic skeletal components such as the pelvis and skull. The purpose of the current study was to systematically evaluate the diagnostic accuracy of sternal measurements for sex estimation and to identify methodological- or population-based moderators that influence classification performance. **Methods**: A systematic review and meta-analysis were conducted in accordance with PRISMA 2020 guidelines. R programming software was used to perform statistical meta-analysis. Pooled sensitivity, specificity, diagnostic odds ratio (DOR), likelihood ratios (LR±), and overall accuracy were calculated using random-effects meta-analysis. Subgroup analyses and meta-regression were performed based on population origin, study design, statistical approach, and measurement protocol. **Results**: Forty-one studies comprising 293 predictive models were included. The overall pooled sensitivity and specificity were 80.9% (95% CI: 79.7–82.1) and 74.0% (95% CI: 72.4–75.5), respectively, with a mean accuracy of 77.3%. Subgroup analysis revealed that studies involving African populations and imaging-based methods achieved the highest accuracy. Machine learning- and ROC-based methods outperformed traditional discriminant analysis. Combined sternal measurements (manubrium and body) yielded the most robust diagnostic performance (accuracy: 87.3%). Significant heterogeneity (I^2^ > 85%) was observed. **Conclusions**: Sternal morphometry exhibits a moderate to high degree of diagnostic accuracy in sex estimation and possesses significant forensic importance, especially in situations where more sexually dimorphic features are inaccessible. Nonetheless, variations across populations, the absence of standardized protocols, and methodological heterogeneity constrain its universal applicability.

## 1. Introduction

Sex estimation in forensic anthropology is irreplaceable, as it corresponds to the first step in constructing the biological profile of unidentified human remains. Accurate biological sex estimation (male or female) follows the estimation of age, stature, and ancestry [1]. Typically, the pelvis is considered the most sexually dimorphic human bone, with accuracy exceeding 95% [2], while the femur and the skull are reliable alternatives [3]. This is not always the case, as in several contexts (mass disasters, criminal cases, or archeological excavations) the pelvis or skull might be missing or fragmented [4,5,6].

Therefore, researchers over the years have investigated alternatives to several landmarks and measurements. Machine learning classifiers of long bones have produced accuracies of 90–92% [7]. Other cranial measurements have been proposed, such as the foramen magnum [8], the sella region [9], orbit [10] and the pterion [11]. These anatomical structures have been traditionally considered with substantial levels of sexual dimorphism, ranging from moderate to high diagnostic accuracy. Nevertheless, dental morphometrics such as permanent canines have also been evaluated [12].

Amid the wide variability in the sex estimation literature, the sternum has been proposed as a reliable and promising element. Anatomically, it is composed of the manubrium, sternal body, and xiphoid process. Other measurable parameters are the sternal index (manubrium divided by the sternal body) and the sternal area [13]. The sternum is often well-preserved due to its compact structure and central location in the thorax. However, in the current literature, a vast number of anatomical measurements and their combinations have been used in forensic studies [13].

Based on these details of the literature, a systematic review and meta-analysis about the diagnostic accuracy of sternal measurements was missing. Specifically, several sternal measurements and statistical analyses are currently being used. Therefore, the purpose of the current study was to conduct such a meta-analysis, combined with meta-regression, on the geographical distribution, the statistical analyses used across the studies, and the measurements used for sex estimation. Ultimately, the identification of the most accurate measurements and the corresponding statistical analysis will maximize the diagnostic accuracy of the sex identification based on the sternum.

## 2. Materials and Methods

### 2.1. Methodology

The current systematic review was performed in accordance with the protocol proposed by the Preferred Reporting Items for Systematic Reviews (PRISMA) 2020 guidelines [14]. According to the guidelines, the protocol was registered in the PROSPERO database with the number CRD420251147325. Moreover, risk of bias assessment for the studies was performed according to the “Checklist for Diagnostic Test Accuracy Studies” proposed by the Joanna Briggs Institute (JBI). According to this tool, the studies were classified as “high risk of bias”, “moderate risk of bias”, and “low risk of bias” based on the percentage of “Yes” answers on the scoring process. Specifically, “low risk” was considered when “Yes” exceeded 70%, “moderate risk” when “Yes” ranged between 50 and 69%, and “high Risk” when “Yes” was lower than 50%. Studies categorized as “High Risk” were subsequently excluded from the quantitative meta-analysis to ensure data integrity [15].

### 2.2. Research Question

The present study was designed to address the following research question: What is the diagnostic accuracy of sternal measurements for sex estimation in the existing literature? To this end, we included studies that applied predictive models to evaluate sex classification based on various sternal morphometric parameters. Only studies reporting explicit accuracy outcomes for biological sex determination were considered eligible. Studies were excluded if the biological sex of the skeletal remains was unknown, predictive modeling was not employed, or the study design involved non-human samples, case reports, or review articles.

### 2.3. Literature Search

Two independent reviewers (IP, DG) performed the literature search and data extraction. Potential disagreements were resolved in discussion with the senior authors (G.T., S.P., and M.P.). Additionally, we have calculated and included the inter-rater reliability (Cohen’s Kappa) to further validate the selection process. The electronic databases PubMed/MEDLINE, Scopus, Web of Science, and Google Scholar were used to search the primary literature through May 2025. The following keywords were used in several combinations and searches: “sternum”, “sternal”, “measurements”, “morphometry”, “sex estimation”, and “sex determination”. There were no date or language restrictions for this process. A secondary literature analysis was conducted by hand-searching the reference lists of all included articles. The eligible studies were gathered, and their data were extracted into Microsoft Excel sheets prior to statistical analysis.

### 2.4. Statistical Meta-Analysis

Statistical analysis was conducted using the open-source R programming language (RStudio version 4.3.2) with the “meta”, “metafor”, and “mada” packages, and the Python programming language (Version 3.12.3) with “matplotlib” for the schematic representation, by a single researcher (GT). The diagnostic accuracy used for sex estimation requires sensitivity and specificity measurements. Sensitivity is the percentage of individuals correctly classified as positive, while specificity is the percentage of individuals correctly classified as negative. For the current study, sensitivity was defined as correctly assigned male, and specificity as correctly assigned female.

For each included study, we constructed 2 × 2 contingency tables of true positives (TP, males correctly classified), true negatives (TN, females correctly classified), false positives (FP, females misclassified as male), and false negatives (FN, males misclassified as female). From these, we calculated the following:

- Sensitivity = TP/(TP + FN).
- Specificity = TN/(TN + FP).
- Diagnostic odds ratio (DOR) = (TP × TN)/(FP × FN).

All accuracy parameters were logit-transformed (for sensitivity and specificity) or log-transformed (for DOR) prior to pooling. Pooled estimates were calculated using random-effects meta-analysis (DerSimonian–Laird method), which accounts for both within- and between-study variance. Back-transformation yielded summary estimates of sensitivity, specificity, and DOR, with 95% confidence intervals (CIs). To provide an overall measure of diagnostic performance, we constructed a summary receiver operating characteristic (sROC) curve using the Moses–Littenberg method.

In addition to these measurements, we also calculated:

- Overall accuracy = (TP + TN)/(TP + TN + FP + FN), representing the proportion of correctly classified individuals.
- Positive likelihood ratio (LR+) = Sensitivity/(1—Specificity), indicating how much more likely a positive test result is to occur in males compared to females.
- Negative likelihood ratio (LR–) = (1—Sensitivity)/Specificity, indicating how much less likely a negative test result is to occur in males compared to females.

Interpretation of LRs followed pre-established thresholds: LR+ > 10 or LR– < 0.1, indicating a large and often conclusive change from pre-test to post-test probability. LR+ 5–10 or LR– 0.1–0.2, corresponds to moderate change. LR+ 2–5 or LR– 0.2–0.5 a small but sometimes important change. LR+ 1–2 or LR– 0.5–1.0 is negligible impact [12].

Cochran’s Q statistics were used to evaluate the presence of heterogeneity across studies, and the Higgins I^2^ statistic was used to quantify heterogeneity. Cochran’s Q *p*-value < 0.10 was considered significant. Higgins I^2^ values between 0 and 40% were regarded as not necessary, 30–60% as moderate heterogeneity, 50–90% as substantial heterogeneity, and 75–100% as considerable heterogeneity.

Lastly, to assess publication bias, the Deeks’ funnel plot asymmetry test was used, regressing the log diagnostic odds ratio against the inverse of the square root of the effective sample size. A *p*-value of less than 0.05 was considered significant across the current meta-analysis.

## 3. Results

### 3.1. Selection Process

**Figure 1:**
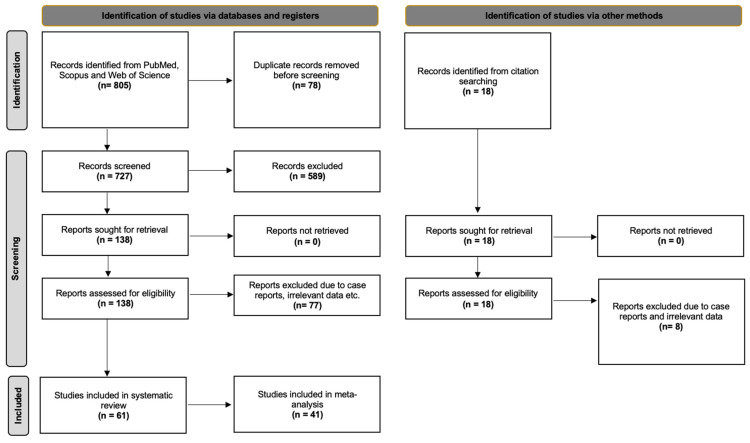
PRISMA 2020 flow chart of literature search analysis.

The databases retrieved 805 results that were exported to Mendeley version 2.10.9 (Elsevier, London). After excluding irrelevant and duplicate papers, 138 studies were retrieved for full-text screening. Finally, 51 studies were considered eligible for our initial meta-analysis questions. Furthermore, 10 studies were identified from references and a hands-on search. Hence, 61 studies were included in the current systematic review. However, during the risk of bias assessment, 20 studies were considered “High” risk of bias and subsequently were excluded from the statistical meta-analysis. Cohen’s Kappa for inter-rater variability was 0.937, indicating excellent agreement. In accordance with the PRISMA 2020 guidelines, Figure 1 presents the flow diagram of the selection process.

### 3.2. Studies Characteristics

The main characteristics of the included studies are presented in Table 1 and Supplementary Materials. A total of 41 studies, comprising 293 predictive models, were eligible for quantitative synthesis. In terms of population origin, 181 models were derived from Asian cohorts, 60 from African cohorts, 42 from European cohorts, and 10 from American cohorts. According to the study design, 182 models were based on imaging techniques, 59 on osteological measurements, and 52 on cadaveric investigations. With respect to the analytical approach, discriminant analysis was the most frequently applied method (*n* = 172). Regarding the variables used for sex estimation, combined measurement models that incorporated the manubrium, sternal body, and sternebrae were the most common (*n* = 84).

### 3.3. Main Meta-Analysis Outcomes

**Figure 2:**
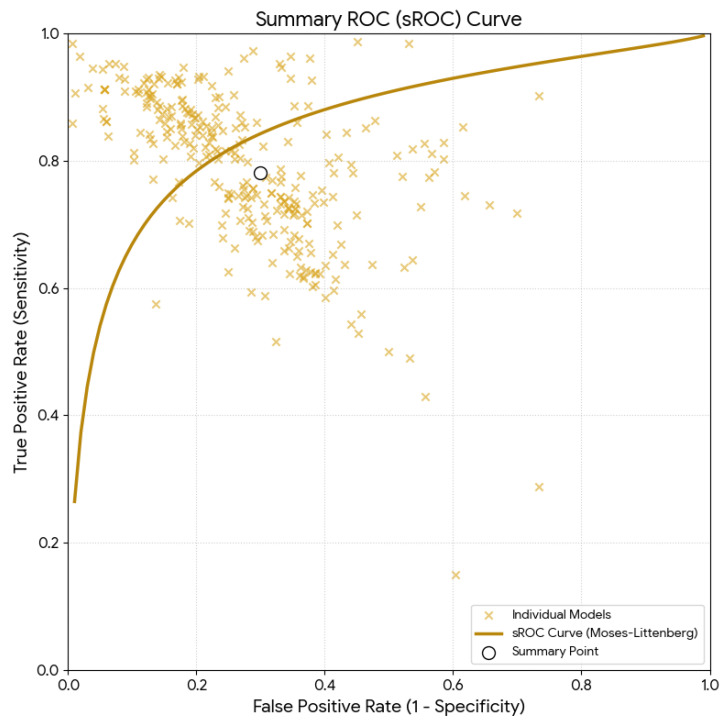
Summary receiver operating characteristic (sROC) curve using the Moses–Littenberg method for the overall estimation.

The analysis of 293 predictive models from 41 studies calculated a pooled sensitivity of 80.94% (95% CI: 79.7–82.1) and a pooled specificity of 73.96% (95% CI: 72.4–75.5). Both values depicted substantial heterogeneity (I^2^ = 85.51% and I^2^ = 87.40%, respectively). The mean accuracy was estimated as 77.3% (95% CI: 76.1–78.5), and it is illustrated in Figure 2 with its sROC curve. The LR+ was 3.01 (95% CI: 2.83–3.20) and the LR- was 0.27 (95% CI: 0.25–0.29), indicating moderate changes in the pre-test-to-post-test probability. Finally, the DOR was calculated as 12.88 (95% CI: 11.23–14.78).

**Figure 3:**
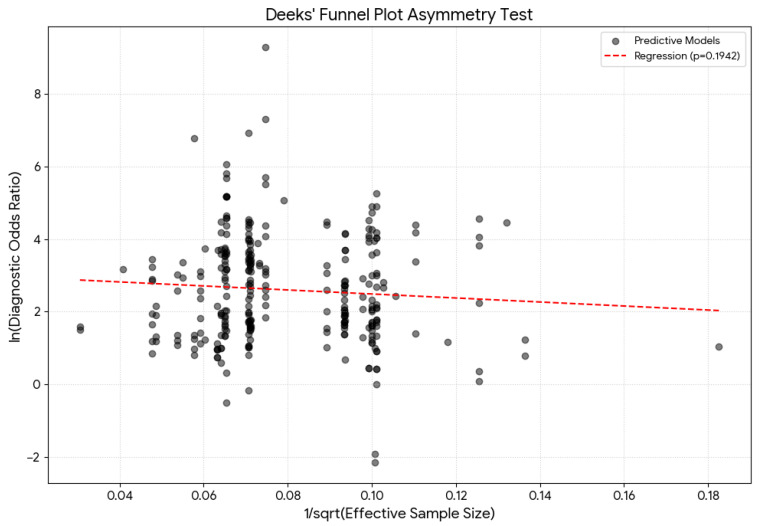
Deeks’ funnel plot test for the overall estimation asymmetry with the associated regression test.

The respective Deeks’ funnel plot is presented in Figure 3. The Deeks’ test yielded a *p*-value of 0.1942, indicating a lack of significant asymmetry in the funnel plot.

### 3.4. Subgroup Analysis

Studies were subgrouped based on nationality (continent), type of study (osteological, imaging, or cadaveric), type of test (discriminant, logistic, ROC, area under the curve, limiting point, or unique algorithm), and sternal measurements used (manubrium, body, xiphoid, sternal area, sternal index, or combinations) (Table 2).

#### 3.4.1. Subgroup Analysis: Nationality

**Figure 4:**
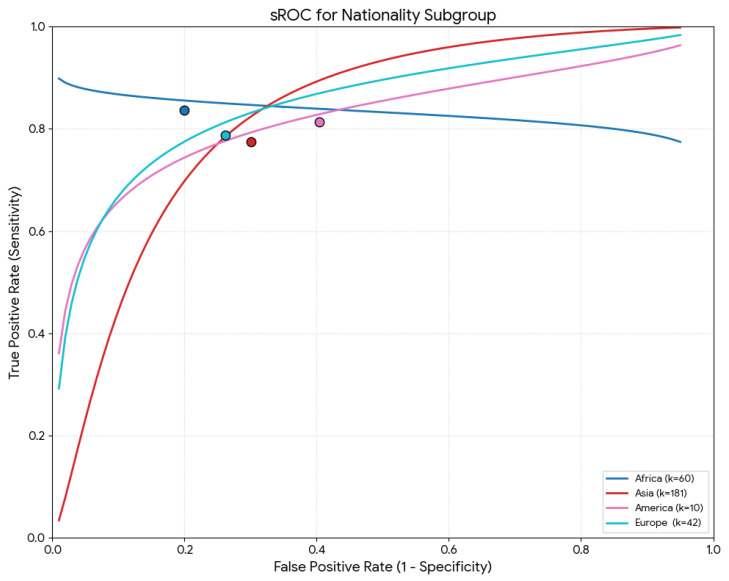
Summary receiver operating characteristic (sROC) curve using the Moses–Littenberg method for the nationality subgroup.

Table 2 summarizes all parameters (sensitivity, specificity, DOR, mean accuracy, and LR+/−) by continent of origin. Regarding the meta-regression results, the global test comparison retrieved no statistical significance for the sensitivity (*p* = 0.472). However, nationality was a statistically significant moderator of specificity and mean accuracy (*p* < 0.001). Specifically, studies with the African population depicted the highest pooled specificity (82.4%) and accuracy (84.2%), while studies from the Asian population had the lowest pooled specificity (59.5%) and accuracy (72.1%) (Figure 4).

#### 3.4.2. Subgroup Analysis: Type of Study

**Figure 5:**
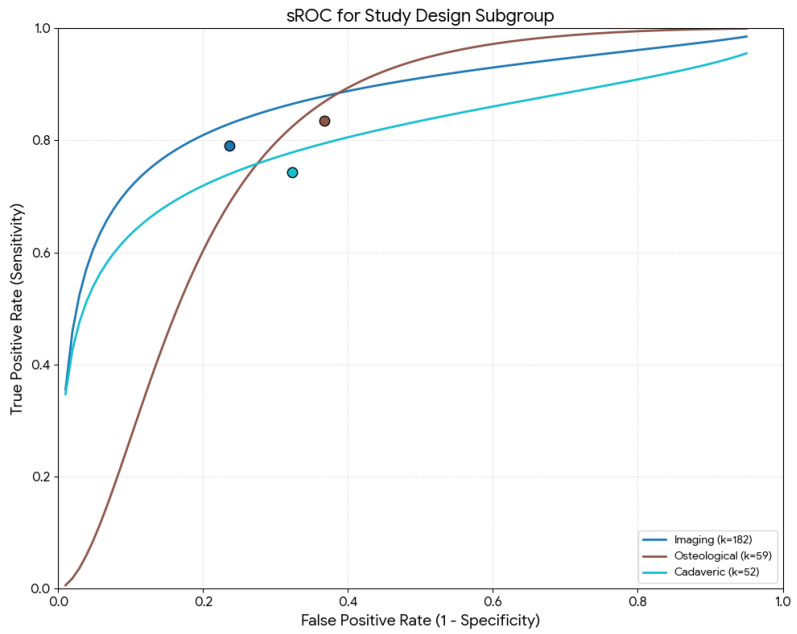
Summary receiver operating characteristic (sROC) curve using the Moses–Littenberg method for the study design.

Table 2 summarizes the parameters (sensitivity, specificity, DOR, mean accuracy, and LR+/−) by study type. Regarding the meta-regression results, the global test comparison found no statistical significance for sensitivity or mean accuracy (*p* = 0.154 and *p* = 0.276, respectively). However, the type of study was a statistically significant moderator for the specificity (*p* = 0.003). Specifically, studies based on imaging techniques had the highest pooled specificity (77.8%), and the osteological studies had the lowest (64.3%) (Figure 5).

#### 3.4.3. Subgroup Analysis: Type of Statistical Analysis

**Figure 6:**
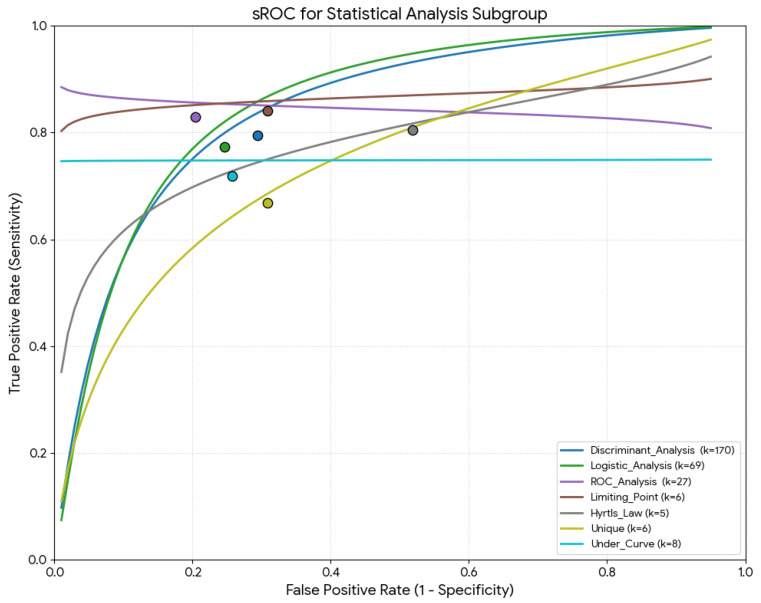
Summary receiver operating characteristic (sROC) curve using the Moses–Littenberg method for the statistical analysis used for the sex estimation.

Table 2 summarizes all parameters (sensitivity, specificity, DOR, mean accuracy, and LR+/−) by statistical analysis type. Regarding the meta-regression results, all global test comparisons were statistically significant (*p* < 0.001). Studies using the rule “136” had the highest sensitivity (95.6%), studies with ROC analysis had the highest specificity (80.9%) and the highest mean accuracy (83.6%) (Figure 6).

#### 3.4.4. Subgroup Analysis: Sternal Measurements

**Figure 7:**
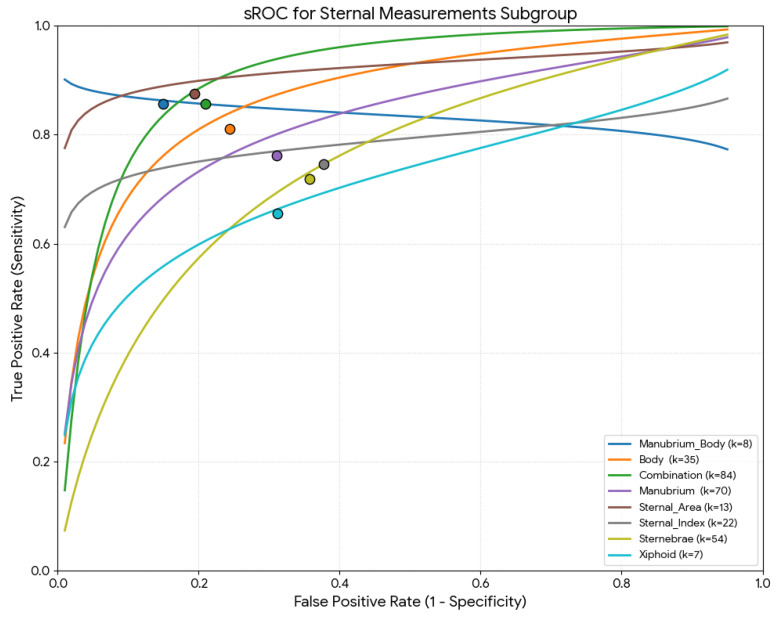
Summary receiver operating characteristic (sROC) curve using the Moses–Littenberg method for the measurements used for the sex estimation.

Table 2 summarizes all the parameters (sensitivity, specificity, DOR, mean accuracy, and LR+/−) based on sternal measurements. Regarding the meta-regression results, all global test comparisons were statistically significant (*p* < 0.001). Studies based on sternal area measurements had the highest pooled sensitivity (88.9%), studies using combined manubrium and sternal body measurements had the highest pooled specificity (86.8%) and accuracy (87.3%) (Figure 7).

## 4. Discussion

The present systematic review and meta-analysis evaluated the diagnostic accuracy of sternal measurements for sex estimation. Analysis of 293 predictive models from 41 studies revealed a pooled sensitivity of 80.9% and a specificity of 74.0%, with an overall accuracy of 77.3%. While sternal morphometry exhibits a theoretical capacity for moderate to high diagnostic accuracy, its practical application is currently circumscribed by significant methodological- and population-based heterogeneity. Our findings suggest that high accuracy (up to 87.3%) is achievable primarily through the adoption of standardized, combined measurement protocols and advanced algorithmic analyses, rather than through generalized or historical linear rules.

### 4.1. Subgroup Interpretations

Subgroup analyses in the present review revealed significant moderators. Studies from African populations achieved the highest pooled specificity and accuracy, whereas those from Asian populations reported the lowest values. These differences may reflect population-specific variation in skeletal dimorphism and reinforce the importance of localized standards. The sternum’s development, including the fusion of its component parts, is highly sensitive to these localized factors, reinforcing the necessity for population-specific forensic standards rather than universal models.

The study type was identified as a statistically significant moderator for specificity, with imaging-based analyses achieving 77.8% compared to 64.3% for traditional osteological approaches. This variability likely stems from the superior resolution and three-dimensional reconstruction capabilities of CT, which enable more precise landmark identification than manual measurements on dry bone. Virtual anthropology techniques reduce the physical handling of the remains [1,8].

The shift toward advanced statistical modeling appears to reduce operator-dependent variability and improve diagnostic utility. Machine learning- and ROC-based analyses yielded a mean accuracy of 83.6%, higher than that of classical discriminant functions at 77.1%. Traditional historical protocols, such as Hyrtl’s Law, demonstrated a notably low accuracy of 59.6%. Advanced classifiers are better equipped to handle the non-linear biological variance. Thus, machine learning methods and ROC-based analyses provided higher accuracy than classical discriminant functions, consistent with recent evidence highlighting the superiority of advanced classifiers in forensic applications [3,7].

Heterogeneity is also driven by the lack of standardized measurement protocols across the global literature. Our findings indicate that multidimensional approaches, specifically combined indices of the manubrium and sternal body, capture dimorphism more effectively than single linear dimensions, achieving a robust accuracy of 87.3%. Conversely, univariate measurements such as XP (65.1%) and SI (70.4%) showed significantly lower reliability. This suggests that isolated bone segments may not adequately reflect the overall dimorphism.

### 4.2. Comparison with Other Skeletal Elements

The pelvis is consistently regarded as the gold standard in sex estimation, achieving accuracies above 95% in both traditional assessments and modern metric or morphometric approaches [1,2]. Geometric morphometric analyses of the pubis reach accuracies exceeding 95%, even when applied to fragmented remains [2]. Deep learning algorithms applied to CT reconstructions of coxal bones have recently achieved near-perfect accuracy, ranging from 97.9% to 99.8% [1]. Long bones represent another reliable alternative, with machine learning models yielding 90–92% accuracy [3].

By contrast, cranial and dental structures generally display lower levels of dimorphism. For instance, the foramen magnum provides moderate diagnostic utility, with accuracy varying between 70 and 90% depending on measurement type [8]. Similarly, the sella turcica demonstrates statistically significant but modest dimorphism [9], while orbital metrics analyzed with machine learning achieve only moderate classification performance (accuracy ~68%) [10]. Teeth, particularly permanent canines, are highly durable but yield accuracies of approximately 77–83% [12].

### 4.3. Forensic Implications

The forensic applicability of sternal measurements lies primarily in their preservation, accessibility, and versatility across different contexts. The sternum is among the most frequently recovered thoracic elements, with preservation rates reported at nearly 60% in skeletal collections [18]. It offers a valuable alternative when highly dimorphic elements such as the pelvis or long bones are missing, fragmented, or otherwise unavailable.

However, important caveats remain, as observed in the current results. First, population specificity is crucial: expression of sexual dimorphism in sternal traits varies substantially across groups, as demonstrated by the current meta-analysis, reinforcing that generalized models are not suitable for universal forensic application. Second, while traditional rules such as Hyrtl’s Law or “the 136 rule” are often cited, their reliability has been inconsistent across studies. Instead, multivariate and machine learning approaches consistently outperform univariate or rule-based methods. Finally, even with high accuracies in controlled studies, heterogeneity and publication bias observed in the present meta-analysis suggest variability in real-world forensic performance.

In practice, sternal morphometry should, therefore, be viewed as a secondary but highly valuable option. When the pelvis or long bones are absent, the sternum can provide robust sex estimates, especially if combined with other thoracic or cranial indicators. Advances in imaging and machine learning will likely increase its utility, enabling forensic anthropologists to leverage clinical CT repositories for model training and validation. In this sense, the sternum not only complements traditional approaches but also expands the forensic toolkit in cases of incomplete or compromised remains.

### 4.4. Strengths and Limitations

This meta-analysis represents the most comprehensive synthesis to date of the diagnostic accuracy of sternal measurements for sex estimation. By pooling results from 41 studies and 293 predictive models, it provides robust estimates of sensitivity, specificity, and diagnostic odds ratios across diverse populations and methodological frameworks. Subgroup analyses also allowed exploration of moderators, including study type, statistical model, measurement protocol, and geographic origin, thereby clarifying sources of heterogeneity and providing valuable insights for forensic practice.

Nevertheless, several limitations must be acknowledged. First, substantial heterogeneity was observed across studies (I^2^ > 85% for sensitivity and specificity), indicating variability in study design, population background, and measurement protocols. Although subgroup analyses identified some moderators (such as imaging versus osteological studies and statistical methods), residual heterogeneity remained unexplained. This may be explained by the use of inconsistent anatomical landmarks and the lack of standardized imaging planes (in CT-based studies), which introduce significant technical noise, complicating direct comparisons and potentially inflating pooled estimates. Moreover, operator-dependent variability remains a concern, especially in manual osteometric and cadaveric studies where inter-observer reliability is not always reported. Even in virtual anthropology, technical variations, such as differences in CT slice thickness, can affect the precision of sternal measurements. Second, measurement protocols were not standardized across studies; some relied on traditional rules (such as Hyrtl’s Law), others on linear dimensions or indices, and more recent work employed CT-derived morphometrics and machine learning. This diversity complicates direct comparisons and may inflate pooled estimates. While machine learning- and ROC-based analyses demonstrated superior performance over traditional methods, many of these models carry an inherent risk of overfitting. These limitations underscore the urgent need for standardized measurement protocols and larger, multicenter datasets to validate sternal morphometry across diverse populations

## 5. Conclusions

The current systematic review with meta-analysis yielded a pooled sensitivity of 80.94%, a pooled specificity of 73.96%, and an accuracy of 77.3%. According to meta-regression analysis, these pooled estimates were significantly influenced by nationality, study type (imaging or osteological), statistical analysis used for estimation (traditional methods or machine learning), and the measurements used (manubrium, body, others, or combination). This meta-analysis provides important pooled evidence, supporting the forensic value of sternal morphometry. From a practical perspective, sternal morphometry exhibits moderate to high diagnostic utility and is of significant forensic importance, especially when more sexually dimorphic skeletal features, such as the pelvis, femur, or skull, are inaccessible or fragmented. However, sternum-based sex estimation must be strictly viewed as a secondary or complementary diagnostic method. Its universal applicability is constrained by substantial interpopulation variations and a current lack of standardized global measurement protocols. Although advanced statistical approaches and imaging techniques, particularly the combined measurement of the manubrium and body, yielded the most robust accuracy (87.3%), these findings are subject to the methodological limitations of the primary literature.

## Figures and Tables

**Table 1:** Characteristics of the included studies.

Author (Year)	Nationality	Total Sample (Male/Female)	Type of Study	Sternal Measurements	Method of Estimation	Reason for Exclusion	Risk of Bias
Abdel Aal et al. (2014) [16]	Africa	160 (80/80)	Imaging	Manubrium Body	Discriminant Analysis	-	Low
Ahmed et al. (2017) [17]	Asia	200 (100/100)	Imaging	Body, Combination, Manubrium, Sternal Area, Sternal Index, Sternebrae	Discriminant Analysis	-	Low
Ahmed et al. (2021) [18]	Africa	180 (87/93)	Imaging	Manubrium	Discriminant Analysis	-	Low
Ali et al. (2021) [19]	Africa	77 (NA/NA)	Imaging	-	Logistic Analysis	No male/female samples provided	High
Ankit et al. (2013) [20]	Asia	100 (45/55)	Osteological	Combination	Rule_136	-	Low
Aragao et al. (2022) [21]	America	30 (15/15)	Cadaveric	Combination	Discriminant Analysis	-	Low
Atal et al. (2008) [22]	Asia	100 (56/44)	Cadaveric	Manubrium, Sternebrae	Logistic Analysis	-	Moderate
Banyeh et al. (2024) [23]	Africa	234 (115/119)	Imaging	Body, Combination, Manubrium, Manubrium Body, Sternal Area, Sternal Index, Sternebrae	Logistic Analysis, Discriminant Analysis	-	Moderate
Baraw et al. (2017) [24]	Asia	100 (50/50)	Osteological	Manubrium, Sternebrae	Discriminant Analysis	-	Moderate
Bedalov et al. (2019) [25]	Europe	128 (73/55)	Imaging	Body, Combination, Manubrium, Sternal Area, Sternal Index, Sternebrae	Discriminant Analysis	-	Moderate
Bongiovanni et al. (2012) [26]	America	410 (285/125)	Osteological	Body, Combination, Manubrium, Sternebrae	Discriminant Analysis	-	Moderate
Chandrakanthn et al. (2014) [27]	Asia	117 (67/50)	Cadaveric	Body, Combination, Manubrium, Manubrium Body, Sternebrae	Logistic Analysis, Discriminant Analysis	-	Moderate
Chowdhuri et al. (2019) [28]	Asia	108 (73/35)	Imaging	Combination, Manubrium	Logistic Analysis	-	Low
Darade et al. (2020) [29]	Asia	300 (150/150)	Cadaveric	Body, Combination, Manubrium, Manubrium Body, Sternebrae	Discriminant Analysis	-	Moderate
Darwish et al. (2017) [30]	Asia	60 (30/30)	Imaging	-	-	No definition of gender accuracy in results	High
Dkhar et al. (2014) [31]	Asia	-	Imaging	-	-	No detailed data analysis	High
Dorado-Fernandez et al. (2021) [32]	Europe	202 (117/85)	Cadaveric	Body, Combination, Manubrium, Sternebrae	Discriminant Analysis	-	Moderate
Ekizoglu et al. (2014) [33]	Asia	443 (241/202)	Imaging	Body, Combination, Manubrium, Manubrium Body, Sternal Index, Sternebrae	Discriminant Analysis	-	Low
Elmansy et al. (2024) [34]	Africa	180 (95/85)	Imaging	Body, Combination, Manubrium, Sternal Index, Sternebrae	ROC Analysis	-	Moderate
Elsayed et al. (2022) [35]	Africa	100 (50/50)	Imaging	Combination	Discriminant Analysis	-	Moderate
Franklin et al. (2012) [36]	Oceania	187 (93/94)	Imaging	Combination	Discriminant Analysis	-	Low
Garcia-Parra et al. (2014) [37]	Europe	105 (NA/NA)	Osteological	-	-	Differing number of subjects	High
Ghorbanlou et al. (2022) [38]	Asia	98 (49/49)	Imaging	Body, Combination, Manubrium, Sternal Area, Sternal Index, Sternebrae, Xiphoid	Discriminant Analysis	-	Low
Gupta et al. (2014) [39]	Asia	100 (66/34)	Cadaveric	Combination	Discriminant Analysis	-	Moderate
Hunnargi et al. (2008) [40]	Asia	115 (75/40)	Osteological	Body, Combination, Manubrium	Limiting Point	-	Low
Hunnargi et al. (2009) [41]	Asia	115 (75/40)	Osteological	Sternal Index	Hyrtls Law	-	Low
Iqbal et al. (2021) [42]	Asia	260 (130/130)	Imaging	-	-	No definition of gender accuracy in results	High
Jaiswal et al. (2019) [43]	Asia	65 (46/19)	Cadaveric	Combination, Sternal Index	Discriminant Analysis, Hyrtls Law	-	Moderate
Jit et al. (1980) [44]	Asia	400 (312/88)	Cadaveric	Combination, Sternal Index	Discriminant Analysis, Hyrtls Law	-	Low
Kalbouneh et al. (2021) [45]	Asia	600 (300/300)	Imaging	Combination	Discriminant Analysis	-	Moderate
Karki et al. (2020) [46]	Asia	105 (62/43)	Imaging	Body, Combination, Manubrium, Sternal Index	Limiting Point, ROC Analysis, Rule 136, Hyrtls Law	-	Low
Kosar et al. (2022) [47]	Asia	237 (113/124)	Imaging	Body, Combination, Manubrium, Sternal Index, Sternebrae	Logistic Analysis, ROC Analysis	-	Low
Macaluso et al. (2010) [48]	Africa	206 (123/83)	Osteological	Body, Combination, Manubrium, Manubrium Body, Sternal Area, Sternal Index, Sternebrae	Logistic Analysis, Discriminant Analysis	-	Moderate
Macaluso et al. (2014) [49]	Europe	116 (65/51)	Imaging	Body, Combination, Manubrium, Sternal Area, Sternal Index, Sternebrae	Discriminant Analysis	-	Moderate
Manoharan et al. (2016) [50]	Asia	-	Cadaveric	-	-	No detailed data analysis	High
McCormick et al. (1985) [51]	America	1133 (698/435)	Osteological	Combination, Sternal Area	Unique	-	Moderate
Mukhopadhyay (2010) [52]	Asia	70 (35/35)	Cadaveric	-	-	No definition of gender accuracy in results	High
Nasab et al. (2024) [53]	Asia	61 (38/23)	Imaging	Combination	Logistic Analysis	-	Moderate
Navadijo et al. (2025) [54]	Europe	280 (140/140)	Imaging	-	-	No definition of gender accuracy in results	High
Oner et al. (2019) [55]	Asia	422 (209/213)	Imaging	Body, Combination, Manubrium, Xiphoid	Unique	-	Low
Peleg et al. (2020) [56]	Asia	212 (141/71) 201 (115/76)	Osteological	Combination	Logistic Analysis	-	Moderate
Puttabanthi et al. (2012) [57]	Asia	79 (57/22)	Osteological	Body, Combination, Manubrium, Sternal Index, Sternebrae	Discriminant Analysis	-	Moderate
Ramadan et al. (2010) [58]	Asia	340 (197/143)	Imaging	Combination, Sternal Area	Logistic Analysis	-	Low
Sharma et al. (2018) [59]	Asia	-	Imaging	-	-	No detailed data analysis	High
Singh et al. (2013) [60]	Asia	-	Cadaveric	-	-	No detailed data analysis	High
Soltani et al. (2020) [61]	Asia	200 (100/100)	Cadaveric	Combination, Manubrium, Sternebrae	Under Curve	-	Moderate
Sravan et al. (2025) [62]	Asia	250 (126/124)	Imaging	Body, Manubrium, Sternal Index, Sternebrae	Discriminant Analysis	-	Moderate
Sweilum et al. (2017) [63]	Asia	261 (159/102)	Imaging	Combination	Logistic Analysis	-	Moderate
Toneva et al. (2014) [64]	Europe	76 (47/29)	Osteological	Sternal Index	Hyrtls Law	-	Moderate
Torimitsu et al. (2015) [65]	Asia	200 (100/100)	Imaging	Body, Combination, Manubrium, Sternal Area, Sternal Index, Sternebrae	Discriminant Analysis	-	Moderate
Tun et al. (2015) [66]	Asia	281 (192/89)	Osteological	Body, Combination, Manubrium, Sternal Area, Sternal Index, Sternebrae	Discriminant Analysis	-	Moderate
Vatzia et al. (2025) [13]	Europe	100 (50/50)	Imaging	Body, Manubrium, Manubrium Body, Sternal Area, Xiphoid	Logistic Analysis	-	Low
Verna et al. (2013) [67]	Europe	-	Imaging	-	-	No detailed data analysis	High
Yonguc et al. (2015) [68]	Asia	95 (65/30)	Cadaveric	Body, Combination, Manubrium	ROC Analysis	-	Moderate
Zhan et al. (2018) [69]	Asia	-	Imaging	-	-	No detailed data analysis	
Zhang et al. (2016) [70]	Asia	225 (143/112)	Imaging	Body, Combination, Manubrium	Discriminant Analysis	-	Moderate
Zhang et al. (2019) [71]	Asia	-	Imaging	-	-	No detailed data analysis	High

**Table 2:** Detailed subgroup analysis of the sensitivity, specificity, DOR, LR+/− and mean accuracy. Sensitivity, specificity, and mean accuracy are presented in frequencies (%), while DOR and LR+/− are presented in their absolute values.

Parameters	Sensitivity (95% CI)	Specificity (95% CI)	DOR (95% CI)	LR+	LR−	Mean Accuracy
**Overall**	80.94 (79.7–82.1)	73.96 (72.4–75.5)	12.88 (11.23–14.78)	3.01	0.27	77.3
***Nationality***						
**Africa** (k = 60)	85.4 (82.9–87.6)	82.4 (79.3–85.1)	29.86 (20.53–43.43)	4.82	0.15	84.2
**Asia** (k = 181)	79.4 (77.7–81)	71.4 (69.4–73.3)	10.21 (8.66–12.04)	2.74	0.29	75.6
**America** (k = 10)	83.1 (79.5–86.2)	59.5 (52.1–66.6)	7.36 (4.69–11.59)	2.04	0.30	72.1
**Europe** (k = 42)	79.7 (76.8–82.3)	74.3 (70.9–77.4)	12.38 (9.02–17.01)	3.07	0.27	77.5
***Type of Study***						
**Imaging** (k = 182)	80.7 (79.1–82.2)	77.8 (76.2–79.4)	15.91 (13.23–19.13)	3.60	0.25	79.5
**Osteological** (k = 59)	84.5 (82.5–86.3)	64.3 (60.9–67.6)	10.71 (8.30–13.82)	2.35	0.24	75.5
**Cadaveric** (k = 52)	77 (73.5–77.8)	68.6 (64–72.9)	7.61 (5.59–10.38)	2.41	0.34	73.3
***Type of Statistical Analysis***						
**Discriminant** (k = 170)	81.2 (79.6–82.7)	72.2 (70.2–74.1)	11.95 (10.02–14.26)	2.87	0.26	77.1
**Logistic** (k = 69)	80 (77.4–82.4)	77.2 (74.2–79.9)	14.08 (10.38–19.09)	3.48	0.26	78.8
**ROC** (k = 27)	84.3 (80.6–87.4)	80.9 (76.4–84.8)	25.75 (15.71–42.21)	4.34	0.20	83.6
**Area Under Curve** (k = 8)	73.9 (62.2–83)	74.4 (64.6–82.3)	9.82 (3.59–26.86)	2.84	0.34	74.4
**Limiting Point** (k = 6)	85.8 (75.8–92)	70.5 (57.4–80.9)	15.16 (4.69–48.97)	2.89	0.20	79.8
**Hyrtl Law** (k = 5)	81.6 (72.5–88.1)	47 (30.4–64.3)	4.64 (2.35–9.18)	1.48	0.35	59.6
**Rule “136”** (k = 2)	95.6 (87.3–98.6)	73.3 (56.6–85.2)	69.09 (20.04–238.13)	3.57	0.06	81.4
**Unique Algorithm** (k = 6)	67.7 (58.3–75.8)	69.1 (60.4–76.7)	4.76 (3.81–5.94)	2.09	0.47	66
***Type of Measurements***						
**Manubrium** (k = 70)	77.7 (75.1–80.2)	69.4 (66.4–72.3)	8.37 (6.56–10.69)	2.53	0.33	73.9
**Body** (k = 35)	82.6 (79.1–85.7)	77.1 (73.7–80.2)	16.67 (11.70–23.75)	3.52	0.23	80.2
**Manubrium and Body** (k = 8)	86.7 (81.7–90.5)	86.8 (79–92)	87.3 (81.3–91.6)	6.43	0.16	87.3
**Sternebrae** (k = 54)	72.6 (70.1–75)	64.4 (61.3–67.4)	4.87 (4.07–5.83)	2.04	0.43	68.5
**Xiphoid** (k = 7)	64.2 (58.1–69.8)	69.5 (60.8–77)	4.63 (2.94–7.30)	2.17	0.43	65.1
**Sternal Index** (k = 22)	75.6 (70.6–80)	65.2 (56.4–73.2)	6.19 (3.89–9.85)	2.04	0.39	70.4
**Sternal Area** (k = 13)	88.9 (84.6–92.1)	83.5 (75–89.6)	44.89 (18.62–108.20)	5.44	0.14	86.2
**Combination** (k = 84)	87.1 (85.4–88.7)	80.7 (78.4–82.9)	30.80 (23.99–39.54)	4.47	0.16	84.1

## Data Availability

Please contact the authors for data requests.

## Supplementary Materials

The following are available online at https://www.mdpi.com/article/10.3390/diagnostics16091255/s1, Supplementary Materials: PRISMA 2020 Checklist.
